# Effect of CFIm68 knockdown on RNA polymerase II transcription

**DOI:** 10.1186/s13104-019-4582-8

**Published:** 2019-09-02

**Authors:** Michael Tellier, Jessica G. Hardy, Chris J. Norbury, Shona Murphy

**Affiliations:** Sir William Dunn School of Pathology, University of Oxford, South Park Roads, Oxford, OX1 3RE UK

**Keywords:** RNA polymerase II, CFIm68, Transcription, Pol II pausing, Cleavage and polyadenylation factors

## Abstract

**Objectives:**

Transcription of eukaryotic protein-coding genes by RNA polymerase II (pol II) is highly regulated at initiation, elongation and termination. Transcription is also coordinated with co-transcriptional processing of the emerging pre-mRNA by capping, splicing, and cleavage and polyadenylation. Polyadenylation (poly(A)) site recognition, which defines the end of the mRNA, relies on the cleavage and polyadenylation (CPA) complex. It was previously observed that knocking-down proteins of the CPA complex affects not only recognition of the poly(A) site but also results in increased pausing of pol II at the beginning of genes. This finding suggests that the CPA complex plays a role in regulating pol II turnover after transcription initiation.

**Data description:**

To explore this possibility, we knocked-down a subunit of the cleavage factor I (CFIm), CFIm68, which is part of the CPA complex and involved in alternative polyadenylation, and performed pol II ChIP-seq in absence or presence of a transcription elongation inhibitor. In addition, we performed pol II ChIP-qPCR on a subset of protein coding genes after knocking down CFIm68.

## Objective

Transcription of a eukaryotic protein-coding gene by pol II requires several steps, including transcription initiation, elongation, and termination. During transcription, co-transcriptional processes such as mRNA capping, splicing, and cleavage and polyadenylation also occur and are required for the production of a mature mRNA. The end of a protein-coding gene is defined by one or more poly(A) sites and recognition of a poly(A) site is essential for the cleavage and polyadenylation of the mRNA [1, 2]. Approximately 85 proteins make up the cleavage and polyadenylation (CPA) complex and are distributed between four multi-subunits complexes that regulate poly(A) site recognition, pre-mRNA cleavage, and polyadenylation [2, 3]. The four complexes are cleavage and polyadenylation specificity factor (CPSF), cleavage stimulation factor (CstF), and cleavage factors I (CFIm) and II (CFIIm) [3]. CFIm is composed of two CFIm25 subunits, which binds the pre-mRNA, and two larger subunits, CFIm59 and CFIm68 [4, 5]. CFIm binds the pre-mRNA 40–50 nt upstream of the poly(A) site but its role in pre-mRNA cleavage remains unclear [6]. However, previous studies have shown a shift towards proximal poly(A) site usage following depletion of CFIm25 or CFIm68 [7–9], suggesting a role of CFIm in promoting distal poly(A) site recognition and longer mRNA 3′UTRs [10].

Some proteins of the CPA complex, including CstF64, CPSF73, and the CPA-associated termination factor Xrn2 have been shown to regulate pol II activity at the beginning and end of the transcription cycle [11, 12]. To determine whether depletion of CFIm also affects pol II pausing and transcription, we used a CRISPR/Cas9 approach to reduce the expression of two subunits of CFIm, CFIm25 and CFIm68 [8, 13], and performed pol II ChIP-seq in the CFIm68KD cell line in absence or presence of an inhibitor of cyclin-dependent kinase (CDK)9, whose activity regulates pol II pause release and entry into productive elongation [14].

## Data description

### Cell culture

HEK293 cells were cultured in Dulbecco’s Modified Eagle’s Medium (DMEM, Sigma) supplemented with 10% fetal bovine serum (FBS, Gibco) and 100 units/ml penicillin + 100 µg/ml streptomycin (Gibco). The CFIm68KD cell lines, and its respective control HEK293 Flp, were previously described [8]. The cell lines were treated prior to ChIP-seq with DMSO or 100 µM 5,6-dichlorobenzimidazone-1-β-d-ribofuranoside (DRB, Sigma) for 30 min (Table 1).

**Table 1:** Overview of data files

Label	Name of data file/data set	File types (file extension)	Data repository and identifier (DOI or accession number)
Data file 1 [20, 21]	293 Flp-In Input	Fastq.gz (raw files), bigwig (processed files)	ENA accession number (fastq.gz): https://identifiers.org/ena.embl:SRX2915405 GEO accession number (bigwig): https://identifiers.org/geo:GSM2666454
Data file 2 [20, 21]	293 Flp-In Pol II	Fastq.gz (raw files), bigwig (processed files)	ENA accession number (fastq.gz): https://identifiers.org/ena.embl:SRX2915406 GEO accession number (bigwig): https://identifiers.org/geo:GSM2666455
Data file 3 [20, 21]	293 Flp-In DRB Input	Fastq.gz (raw files), bigwig (processed files)	ENA accession number (fastq.gz): https://identifiers.org/ena.embl:SRX6095228 GEO accession number (bigwig): https://identifiers.org/geo:GSM3898200
Data file 4 [20, 21]	293 Flp-In DRB Pol II	Fastq.gz (raw files), bigwig (processed files)	ENA accession number (fastq.gz): https://identifiers.org/ena.embl:SRX6095229 GEO accession number (bigwig): https://identifiers.org/geo:GSM3898201
Data file 5 [20, 21]	68KD Input	Fastq.gz (raw files), bigwig (processed files)	ENA accession number (fastq.gz): https://identifiers.org/ena.embl:SRX2915407 GEO accession number (bigwig): https://identifiers.org/geo:GSM2666456
Data file 6 [20, 21]	68KD Pol II	Fastq.gz (raw files), bigwig (processed files)	ENA accession number (fastq.gz): https://identifiers.org/ena.embl:SRX2915408 GEO accession number (bigwig): https://identifiers.org/geo:GSM2666457
Data file 7 [20, 21]	CFIm68KD DRB Input	Fastq.gz (raw files), bigwig (processed files)	ENA accession number (fastq.gz): https://identifiers.org/ena.embl:SRX6095230 GEO accession number (bigwig): https://identifiers.org/geo:GSM3898202
Data file 8 [20, 21]	CFIm68KD DRB Pol II	Fastq.gz (raw files), bigwig (processed files)	ENA accession number (fastq.gz): https://identifiers.org/ena.embl:SRX6095231 GEO accession number (bigwig): https://identifiers.org/geo:GSM3898203
Additional file 1 [22]	List of primers	.docx	10.6084/m9.figshare.9159869
Additional file 2 [23]	Pol II ChIP-qPCR results	.pdf	10.6084/m9.figshare.9159878

### ChIP-qPCR and ChIP-seq

ChIP was performed as previously described [15]. Briefly, cells were crosslinked at room temperature with 1% formaldehyde and quenched with 125 mM glycine for 5 min. Nuclear extracts were sonicated twice on a Bioruptor (Diagenode) for 15 min at high amplitude, 30 s ON/30 s OFF. 80 μg of chromatin was incubated overnight at 4 °C with 2 μg of an antibody against IgG (sc-2027, Santa Cruz) or against pol II (sc-899X, Santa Cruz). After recovery of immune complexes with BSA-saturated protein G Dynabeads and extensive washes, crosslinks were reversed by incubation at 65 °C for 5 h. After ethanol precipitation and proteinase K treatment, DNA was purified using a MinElute PCR Purification Kit (Qiagen). A single replicate of ChIP samples were sequenced on an Illumina HiSeq 4000 with 75 bp paired-end reads (Wellcome Trust Centre for Human Genetics, University of Oxford). For ChIP-qPCR, the list of primers can be found in Additional file 1. Pol II ChIP-qPCR were done in biological triplicates and can be found in Additional file 2. Statistical test: unpaired *t* test, *p < 0.05, **p < 0.01, ***p < 0.001.

### Bioinformatics analysis

Adapters were trimmed with Cutadapt v. 1.9.1 [16] with the following constant parameters: --minimum-length 10 –q 15, 10 --max-n 1. Obtained sequences were mapped to the human hg19 reference sequence with Bowtie2 v. 2.2.5 [17]. Unmapped reads were removed with SAMtools v. 1.3.1 [18]. Mapped reads were then de-duplicated using Picard to remove PCR duplicates. Bam files were sorted and indexed with SAMtools. Bigwig files were created with a FPKM (Fragments per kilobase per million mapped reads) normalization by employing deepTools2 v. 2.2.4 [19] bamCoverage tool with the following parameters: -bs 10 -normalizeToRPKM -e –p max. Metaprofiles were created with deepTools2 computeMatrix tool.

## Limitations

The knockdown of CFIm68 was not complete and may therefore may not be sufficient to completely abrogate the role of CFIm68 in pol II pausing and transcription regulation. The ChIP-seq were performed only once and in only one cell line; HEK293. We also performed pol II ChIP-qPCR on a limited number of protein-coding genes.

## Supplementary information

**Additional file 1.** List of primers, List of primers used for the ChIP-qPCR presented in additional file.

**Additional file 2.** Pol II ChIP-qPCR results, results of the pol II ChIP-qPCR on three genes in the HEK293 Flp-In and CFIm68KD cell lines.

## Data Availability

The data described in this Data note can be freely and openly accessed on the GEO website under the Accession Number: GSE99955 [20] and in ENA under the Accession Number PRJNA390279 [21]. Please see Table 1 and reference list for details and links to the data.

## Abbreviations

Pol II: RNA polymerase II
DRB: 5,6-dichlorobenzimidazone-1-β-d-ribofuranoside
ChIP: chromatin immunoprecipitation
DMEM: Dulbecco’s Modified Eagle’s Medium
FBS: fetal bovine serum
FPKM: fragments per kilobase per million mapped reads
3′UTR: 3′ untranslated region
CDK9: cyclin-dependent kinase 9
CPA: cleavage and polyadenylation complex
CFIm: cleavage factor I
