# Supplementary material for: Effect of CFIm68 knockdown on RNA polymerase II transcription
Source: BMC Res Notes. 2019 Sep 2;12:554. doi: 10.1186/s13104-019-4582-8 (PMC6720987; doi:10.1186/s13104-019-4582-8)
Supplement: Supplementary file 1 — Additional file 1. List of primers, List of primers used for the ChIP-qPCR presented in additional file. [file 13104_2019_4582_MOESM1_ESM.docx]

| **Name** | **Forward primer** | **Reverse primer** |
| --- | --- | --- |
| NEG | TGGTACAACCACAGCTCAGTG | AAGCTGGACATGGTTGTGTG |
| KPNB1 TSS | TTACTTCCTCCCTCCAAATGGG | ACAGCCTCCCTTCCTTCTTTC |
| KPNB1 TSS+5.1 | GCAAAGCAGGATACTAAGTGATCCGA | GAAGCCACAATTGACCTAGAGC |
| KPNB1 TSS+20.2 | TGCAAGAGCCAGTGGGAACACTT | CCTCTACTCAGCAATGATACTTC |
| KPNB1 pA-4.8 | CTGAGGAAACTGAAGAACCAAG | GAAGGCAGTGCTTGCCAGAAT |
| KPNB1 pA-2.9 | GAGGAGTGTGCACGGATGCTGAA | CCAAGATGGCCGATGTTATGG |
| KPNB1 pA-0.4 | TAGTTACCGTCTGCTTGGGAAGATG | CCTCTGACAGCAAGTCCAACATT |
| KPNB1 pA+1.4 | GACTCATCACACCAAGGTCAC | GATAGTGCTGGGAAGGAAATGG |
| KPNB1 pA+2.6 | GTACATCTCAGCTTTGGCATATG | GCCCAGAACATAGCAGGCATTGC |
| KPNB1  pA+4.1 | GTTTCACCGTGTTAGCCAGGATGG | CCACAGCCATGTTCATTTCTGC |
| LDLR  TSS | AATCACCCCACTGCAAACTC | TAGCTGGAAACCCTGGCTTC |
| SLCO4A1  TSS | CCAGCGGATGAATGAAGCG | ACTCACCGTCCCCGTCTC |
