# Supplementary figures and images for: Effect of CFIm68 knockdown on RNA polymerase II transcription
Source: BMC Res Notes. 2019 Sep 2;12:554. doi: 10.1186/s13104-019-4582-8 (PMC6720987; doi:10.1186/s13104-019-4582-8)

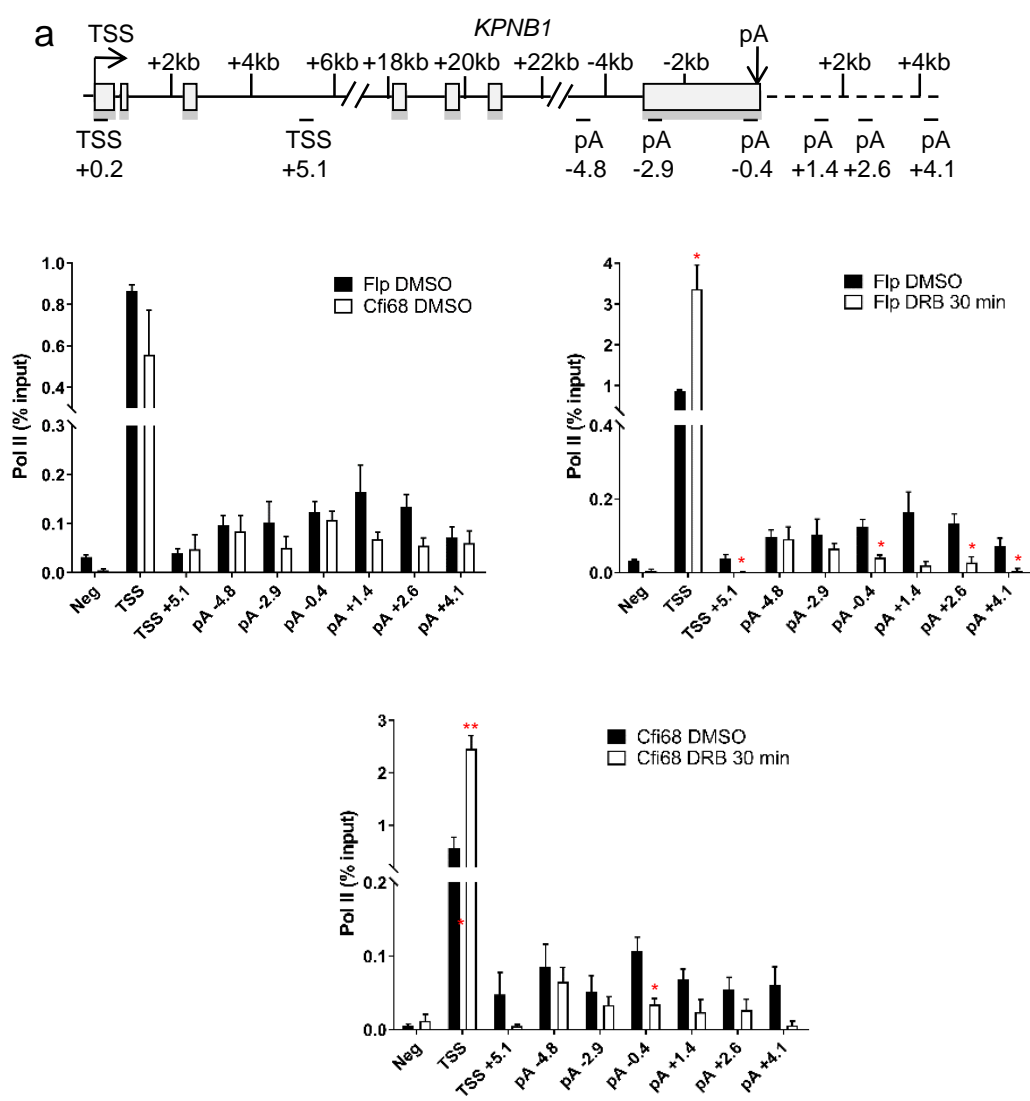

**b**

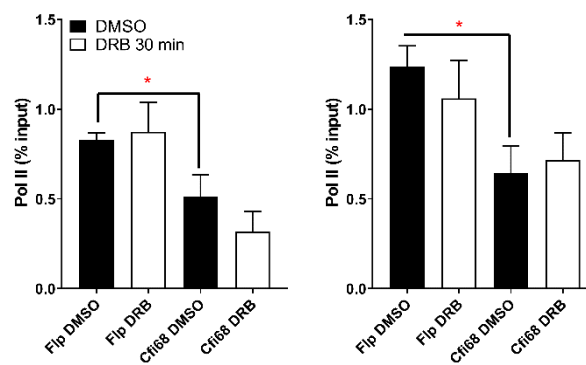

Supplement: Supplementary file 2 — Additional file 2. Pol II ChIP-qPCR results, results of the pol II ChIP-qPCR on three genes in the HEK293 Flp-In and CFIm68KD cell lines. [file 13104_2019_4582_MOESM2_ESM.pdf]
